# Tools to Assess the Trustworthiness of Evidence-Based Point-of-Care Information for Health Care Professionals: Systematic Review

**DOI:** 10.2196/15415

**Published:** 2020-01-17

**Authors:** Gerlinde Lenaerts, Geertruida E Bekkering, Martine Goossens, Leen De Coninck, Nicolas Delvaux, Sam Cordyn, Jef Adriaenssens, Patrick Vankrunkelsven

**Affiliations:** 1 Belgian Centre for Evidence Based Medicine (CEBAM) Leuven Belgium; 2 Department of Public Health and Primary Care Katholieke Universiteiti Leuven Leuven Belgium; 3 Artevelde Hogeschool Ghent University Association Ghent Belgium; 4 Federation of the White and Yellow Cross of Flanders Brussels Belgium; 5 Belgian Health Care Knowledge Centre Brussels Belgium

**Keywords:** evidence-based medicine, evidence-based practice, point-of-care systems, health care quality, internet information, information science, systematic review

## Abstract

**Background:**

User-friendly information at the point of care should be well structured, rapidly accessible, and comprehensive. Also, this information should be trustworthy, as it will be used by health care practitioners to practice evidence-based medicine. Therefore, a standard, validated tool to evaluate the trustworthiness of such point-of-care information resources is needed.

**Objective:**

This systematic review sought to search for tools to assess the trustworthiness of point-of-care resources and to describe and analyze the content of these tools.

**Methods:**

A systematic search was performed on three sources: (1) we searched online for initiatives that worked off of the trustworthiness of medical information; (2) we searched Medline (PubMed) until June 2019 for relevant literature; and (3) we scanned reference lists and lists of citing papers via Web of Science for each retrieved paper. We included all studies, reports, websites, or methodologies that reported on tools that assessed the trustworthiness of medical information for professionals. From the selected studies, we extracted information on the general characteristics of the tools. As no standard, risk-of-bias assessment instruments are available for these types of studies, we described how each tool was developed, including any assessments on reliability and validity. We analyzed the criteria used in the different tools and divided them into five categories: (1) author-related information; (2) evidence-based methodology; (3) website quality; (4) website design and usability; and (5) website interactivity. The percentage of tools in compliance with these categories and the different criteria were calculated.

**Results:**

Included in this review was a total of 17 tools, all published between 1997 and 2018. The tools were developed for different purposes, from a general quality assessment of medical information to very detailed analyses, all specifically for point-of-care resources. However, the development process of the tools was poorly described. Overall, seven tools had a scoring system implemented, two were assessed for reliability only, and two other tools were assessed for both validity and reliability. The content analysis showed that all the tools assessed criteria related to an evidence-based methodology: 82% of the tools assessed author-related information, 71% assessed criteria related to website quality, 71% assessed criteria related to website design and usability, and 47% of the tools assessed criteria related to website interactivity. There was significant variability in criteria used, as some were very detailed while others were more broadly defined.

**Conclusions:**

The 17 included tools encompass a variety of items important for the assessment of the trustworthiness of point-of-care information. Overall, two tools were assessed for both reliability and validity, but they lacked some essential criteria for the assessment of the trustworthiness of medical information for use at the point-of-care. Currently, a standard, validated tool does not exist. The results of this review may contribute to the development of such an instrument, which may enhance the quality of point-of-care information in the long term.

**Trial Registration:**

PROSPERO CRD42019122565; https://www.crd.york.ac.uk/prospero/display_record.php?RecordID=122565

## Introduction

Evidence-based medicine is one of the cornerstones of high-quality health care. This conscientious, explicit, and judicious use of current best evidence in making decisions about the care of individual patients [1] should be facilitated to ensure effective and efficient patient care. With a continuously increasing body of scientific evidence, it is not feasible for health care professionals to access and review the best evidence themselves regularly and independently. Furthermore, they have little time to process large quantities of information during their consultation with patients [2]. Therefore, health care professionals need good quality information that is also user-friendly. This type of information is labeled point-of-care information [3,4], and it is well-structured, rapidly accessible, and comprehensive information for use at the specific point in the workflow when health care professionals and patients interact [3].

Health care professionals routinely use clinical guidelines as reliable sources of information to support their clinical decision-making. Guidelines are statements that include recommendations that are intended to optimize patient care, and that are informed by a systematic review of evidence and an assessment of the benefits and harms of alternative care options [5]. This combination of an assessment of quality of evidence and the benefits and harms means guidelines are most suited to guide clinical decision-making. Furthermore, a validated instrument is available to assess the quality of guidelines [6]. This instrument, known as AGREE II (Appraisal of Guidelines for Research and Evaluation), was developed for the assessment of the validity and trustworthiness of clinical guidelines and is nowadays recognized as an international standard. The use of such an instrument enhances the quality of guidelines [7,8]; however, for many clinical problems or health care professions, there are no or limited guidelines available. In that case, one depends on other information sources. Thanks to the internet, a vast amount of information is accessible within a few mouse clicks, but identification of the most relevant information and assessment of its quality and transparency is indispensable when used in clinical practice. Although different instruments for assessment of the methodological quality of systematic reviews [9-11] or individual studies [12,13] do exist, these instruments are not appropriate for evaluation of the trustworthiness of point-of-care information. Banzi et al [3] reviewed online point-of-care information summary providers. They developed a tool to evaluate the breadth, content development, and editorial policy against their claims of being “evidence-based.” However, this tool was never tested on validity and reliability.

We aimed to search for a valid tool to assess the trustworthiness of point-of-care information. To this end, we performed a systematic review to identify existing tools and examined their validity and reliability.

## Methods

### Overview

We performed a systematic review using the standards for systematic reviewing reported by Cochrane [14], and the Preferred Reporting Items for Systematic Reviews and Meta-Analyses (PRISMA) guidelines were used for the reporting of our findings [15]. The protocol of this review was registered at PROSPERO (CRD42019122565).

### Search Strategy

To identify tools, we used three sources of information. First, we searched the internet for institutes or initiatives that worked on the trustworthiness of health information. Second, we searched Medline (via PubMed) for relevant literature. A search from the inception of the database to June 2019 was conducted to identify the studies of interest. A search string was built using the concepts of trustworthiness and point-of-care information (Textbox 1). Terms within a concept were combined using the Boolean operator ‘OR.’ Then, the terms between the concepts were combined with the Boolean operator ‘AND.’ Lastly, we scanned the reference lists and lists of all citing papers via Web of Science for each retrieved paper, to identify additional tools that were not found in the previous searches.

**Concepts used to build the search string.**

**Concept ‘trustworthiness.’**

- Mesh-terms: methods; standards (subheading); healthcare evaluation mechanisms; evaluation studies as topic; health care quality, access, and evaluation; reproducibility of results
- Free-text words: methodological quality, quality standards, evidence-based methodology, editorial quality, evaluation, validity, reliability

**Concept ‘point-of-care.’**

- Mesh-terms: health information systems; point-of-care systems; medical informatics, consumer health informatics;
- Free-text words: (web-based or electronic or online or internet) and health information; e-Health, e-Health information, point-of-care services, point-of-care information

### Inclusion and Exclusion Criteria

We included all studies, reports, websites, or methodologies that reported on tools, including checklists and criteria, to assess the trustworthiness of medical information for health care professionals. We used the following criteria:

1. Tools had to evaluate point-of-care information or resources for professionals. The definition of point-of-care information was web-based medical compendia specifically designed to deliver predigested, rapidly accessible, comprehensive, periodically updated, and evidence-based information (and guidance) to clinicians [3]. We excluded tools to assess the quality of information for patients, as well as tools that assessed the quality of systematic reviews or other primary studies.
2. Tools had to evaluate trustworthiness. Trustworthiness represented features that made users trust the information, including methodological quality and editorial transparency. The tools that only assessed user-friendliness were excluded.
3. Tools had to be published by multiple authors or an organization.
4. Tools had to be freely available. In the case of websites that contained multiple tools, they were separated by their methodology.
5. Additionally, we excluded tools to assess the quality of mobile applications.

### Selection of Articles and Web Pages

Tools were selected by two researchers (GB, GL) independently. The selection of journal articles was made in two steps: (1) all titles and abstracts were compared against the selection criteria; and (2) the full texts of potential eligible articles were retrieved and subsequently compared against the inclusion and exclusion criteria. The two researchers resolved discrepancies in selection by discussion and consensus.

### Assessment of Methodological Quality

To date, there are no standards to assess the methodological quality of tools to determine the trustworthiness of point-of-care information. Therefore, we could not perform a standard risk-of-bias assessment on each tool. However, we checked each tool for potential risk of bias in the developmental phase, including looking for a lack of validity and performing a reliability assessment. Also, we extracted details on the development of the tools.

### Data Extraction and Analysis

A data overview table was used to extract data from the available tools (Textbox 2). We noted the general characteristics and described the purpose for which a tool was developed and the criteria and scoring systems used. The data extraction was then performed by one researcher (GL) and checked by a second researcher (GB). Discrepancies were identified and resolved through discussion. Based on the data overview table, both the similarities and differences of the characteristics of the tools used to assess the trustworthiness of point-of-care information were analyzed. To examine possible overlap between tools, we listed all criteria and mapped them into general ones, then divided those into five main categories: (1) author-related information; (2) evidence-based methodology; (3) website quality; (4) website design and usability; and (5) website interactivity (see Multimedia Appendix 1). Based on these categories and criteria, we described the characteristics of the tools using descriptive statistics.

**Data overview table.**

**Characteristics of the tool**

- Name
- Aim
- Developer
- Is there a sum score of final combined judgment?
- Description (items, elements)
- Description (scoring method)
- Remarks

**Development of the tool**

- How was the tool developed? (descriptive)
- For which purpose was the tool developed? (descriptive)
- Was the tool assessed for validity? (Y/N)
- Was the tool assessed for reliability? (Y/N)
- Other relevant details (descriptive)

## Results

### Search Strategy

**Figure 1:**
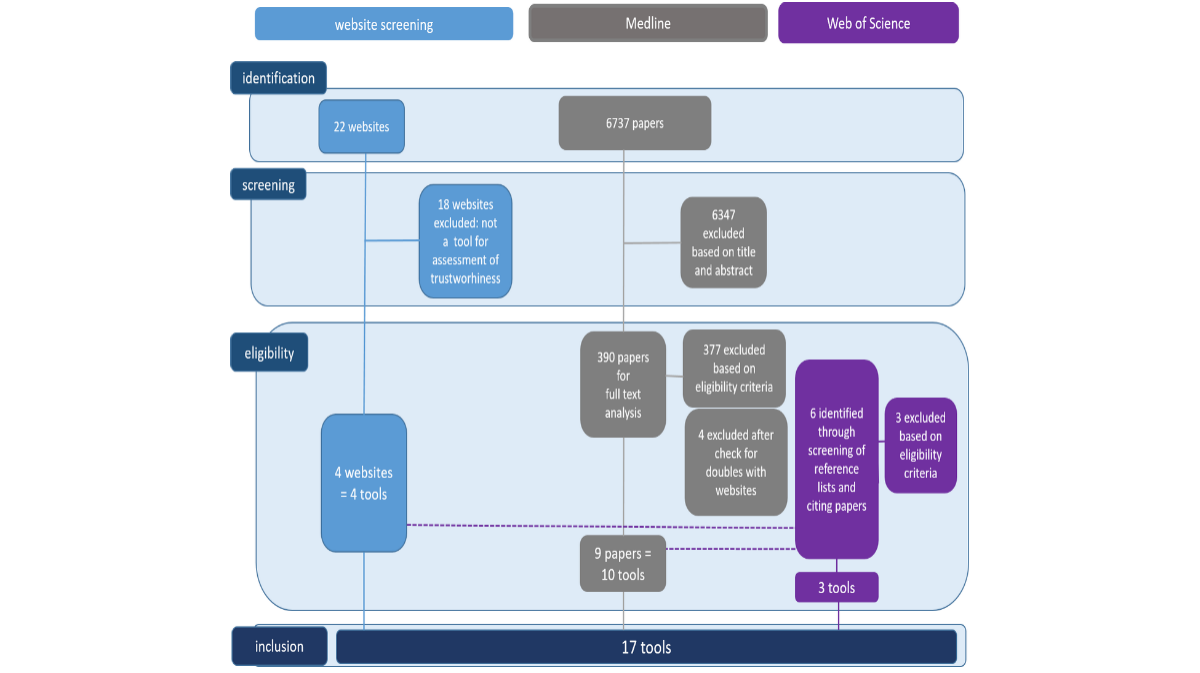
Search strategy.

The flow chart shown in Figure 1 summarizes the results of the systematic search. After identification of relevant websites and titles and screening of abstracts and full texts for eligibility, 16 papers that reported on tools or criteria to assess the trustworthiness of point-of-care information used by health care professionals were included. One study reported on more than one tool [16]. Finally, 17 tools were included in this review.

### General Characteristics of the Tools

Table 1 provides an overview of the general characteristics of the included tools. All tools were developed between 1997 and 2018, and they originated from the United States, Canada, Europe, Switzerland, Singapore, and Iran. The Health on the Net (HON) code [17] and the electronic health (eHealth) Code of Ethics [18] are both codes of conduct and the result of international collaboration. They were developed based on discussion and consensus with international expert panels and underwent peer review. The HONcode aims to control the quality of health information on the internet and provides a quality seal for HONcode-certified websites [17]. The eHealth Code of Ethics provides guiding principles to understand the risk and potential of health information on the internet for professionals, producers, and consumers, and aims to contribute to high-quality information in this way [18].

The Silberg [19], Kapoun [20], Gillois [21], Jiang criteria [22], and CART (Completeness, Accuracy, Relevance, Timeliness) [23] tools aimed to critically appraise and evaluate the quality, credibility, and appropriateness of health information on the internet. The development process of these tools was poorly described. The authors relied on existing criteria for the quality assessment of the information [20-23], or the critical thinking process of the authors was the basis for the definition of the criteria [19]. Likewise, the Sandvik scale [24], QUEST (Quality Evaluation Scoring Tool) [25], and the 11-Point Quality Assessment Scale [26] were developed for the same purpose, but they have a scoring system implemented. The Aslani criteria [16] are based on the HONcode, Silberg criteria, Kapoun criteria, Sandvik scale, and the Health Information Technology Institute (HITI) criteria. However, these criteria were excluded from this review because they are not available anymore.

The AMA (American Medical Association) developed principles [27] as guidelines meant to apply to all AMA websites, but they were also intended to guide the creators of websites that provide medical information for professionals and consumers. The principles are developed and regularly reviewed by AMA staff members and an external advisory panel of experts.

The Grid ULiège [28,29] and the Trumble Tool [30] are the most comprehensive tools and were developed for the analysis and evaluation of medical websites. An Excel-based evaluation form allows the calculation of a final, weighted score based on 38 or 23 items, respectively. The Trumble tool was specifically developed to evaluate evidence-based medical tools at point-of-care. The Banzi tool [3] aims to evaluate and score the breadth, content development, and editorial policy for point-of-care summaries against their claims of being evidence-based. Finally, OncoRX-IQ [31] is a tool developed for the assessment of the quality of information of online drug databases for anticancer drug interactions. The Banzi tool [3], the Trumble tool [26], and the 11-Point Quality Assessment Scale [30] are the only tools that specify the evaluation of evidence-based principles of online health information. The definitions of the content of these tools were done by researchers who arbitrarily postulated criteria that, to their opinion, would best describe the quality of point-of-care information. Only 4/17 (24%) tools were assessed for reliability [21,25,31] and only 2/17 (12%) for validity [25,26] (Table 1).

**Table 1:** Characteristics of the tools.

Name of tool	Language	Date of publication	Country of origin	Number of items	Assessed for reliability or validity	Scoring system
Silberg criteria [19]	English	1997	United States	4	—	—
HONcode^a^ [32]	English	1998	Based in Switzerland, international working group	8	—	—
Kapoun criteria [20]	English	1998	United States	5	—	—
Sandvik scale [24]	English	1999	Norway	7	—	Yes
Gillois criteria [21]	English	1999	France	9	Reliability	—
Joubert criteria [33]	English	1999	France	8	—	—
AMA^b^ principles [27]	English	2000	United States	14	—	—
eHealth^c^ Code of Ethics [18]	English	2000	United States, international working group (WHO^d^/PAHO^e^)	17	—	—
Jiang criteria [22]	English	2000	United States	7	—	—
Grid ULiege [28,29]	English	2003	Belgium	38	—	Yes
CART^f^ [23]	English	2006	United Kingdom	4	—	—
Trumble Tool [30]	English	2006	United Kingdom	23	—	yes
Banzi tool [3]	English	2010	Italy	10	—	yes
OncoRx-IQ [31]	English	2010	Singapore	19	Reliability	yes
11 Point Quality Assessment Scale [26]	English	2012	Canada	11	Reliability and validity	yes
Aslani criteria [16]	English	2014	Iran	10	—	—
QUEST^g^ criteria [25]	English	2018	Canada	6	Reliability and validity	yes

^a^HON: health on the net.

^b^AMA: American Medical Association.

^c^eHealth: electronic health.

^d^WHO: World Health Organization.

^e^PAHO: Pan American Health Organization.

^f^CART: Completeness, Accuracy, Relevance, Timeliness.

^g^QUEST: Quality Evaluation Scoring Tool.

### Content Analysis of the Tools

Multimedia Appendix 1 presents an overview of the 17 included tools with their criteria for the assessment of the trustworthiness of point-of-care information. Altogether, the tools cover 156 criteria. These were combined into 36 general criteria, mapped in five main categories: (1) author-related information with 4 related criteria; (2) evidence-based methodology with 15 related criteria; (3) website quality with 8 related criteria; (4) website design and usability with 7 related criteria; and (5) website interactivity. Some criteria described in the tools were broad and covered more than one general criterion and vice versa, whereas some criteria described in the tools were detailed and therefore summarized in one general criterion. For a few tools [3,18,27,28,30,31], we excluded some of the criteria because they were inappropriate for the assessment of trustworthiness or not applicable in the current context.

Multimedia Appendix 2 presents the prevalence of criteria in the 17 included tools. Overall, 14/17 tools (82%) of the tools addressed author-related information. Only the Joubert criteria, CART, and the 11-Point Quality Assessment Scale did not assess author-related information. All 17 tools (100%) addressed one or more items in the category of evidence-based methodology. The criteria “references to source data” (n=11; 65%) and “content is current and actual” (n=15; 88%) were the most frequently assessed in this category. A total of 12 tools (71%) assessed criteria related to website quality. The most frequently assessed criteria in this category were “transparent ownership” (n=9; 53%) and “financial information” (n=9; 53%). Website design and usability were evaluated by 12 tools (71%). The criterion “ease of use and navigation” (n=11; 65%) was the most frequently used. Website interactivity refers to functions that allow contact or discussion with the authors or site owners. This category was mentioned in 8 tools (47%).

### Assessment of Reliability and Validity of Tools

The reliability and validity of the tools were scarcely reported. Interrater reliability was calculated by kappa coefficients [25,26], Kendall coefficients [31], or by calculation of a percentage of agreement between two researchers [21]. QUEST was compared to three other criteria-related tools to calculate convergent validity. The quality scores generated by each pair of tools were compared to calculate Kendall tau-ranked correlation. The 11-Point Quality Assessment Scale stated that the tool was previously validated, but no information on the validation process could be found.

## Discussion

### Primary Findings

This review studied 16 articles that reported on 17 tools analyzing the trustworthiness of point-of-care information. Our main finding was that the trustworthiness of information is currently assessed and scored in different ways, as illustrated by essential differences in the number of criteria and the content addressed by the tools. This reveals the need for consistency and completeness in evaluating the quality of health information resources. Therefore, this review extends the current literature by giving an overview of existing tools, including their criteria and general characteristics.

To assess the trustworthiness of health care information, we need reliable tools that have been assessed on reliability and validity. Only QUEST [25] and the 11-Point Quality Assessment Scale [26] were assessed on both reliability and validity. However, QUEST only encompasses criteria on author-related information and evidence-based methodology and is therefore too concise for quality assessment of point-of-care information. The 11-Point Quality Assessment Scale was developed as a quality measure for online texts and covers criteria related to evidence-based methodology and usability. However, criteria for the assessment of author-related information and website quality, such as transparent ownership and financial disclosures, were missing, and the validation process was not described. The absence of reliable and validated tools is an essential finding of this review and a shortcoming in the field.

The criteria used in tools to assess the trustworthiness of medical information showed much variation. We encountered this variation when it became apparent that it would be difficult to structure all the original criteria and to reformulate general criteria. Some criteria overlapped with others, using slightly different terms, which illustrated the lack of uniformity and consistency in tools for quality assessment of point-of-care information.

Back in 2001, Risk and Dzenowagis [34] highlighted the complexity of health information on the internet and analyzed the major quality initiatives. A set of quality criteria for health information and credible enforcement tools were named as essential elements for successful quality programs. Nowadays, the need for a uniform, validated tool for the quality assessment of point-of-care resources is still present. Currently, the quality of most point-of-care information is low [3,35]. Risk has suggested tool-based evaluation of quality and third-party certification of compliance as critical mechanisms for quality improvement [34]. A valid tool may improve the quality of point-of-care information, as was also reported for guidelines [8].

### Content of the Tools

All the tools have criteria to assess the evidence-based methodology used to summarize the information. Some use only two [16,19,27] while other tools have seven or more criteria in this category [3,18,26,28,29]. Perhaps the content of the items is more important than the number. For example, “reference to source data” and “content is current and actual” are frequently used, but often these criteria do not guarantee that an information source is truly evidence-based. Remarkably, only a few criteria fit the first three steps in evidence-based medicine: asking a good question, finding the best evidence, and appraising the evidence [36]. For example, “systematic reviews are preferred on primary studies,” “formal grading of evidence,” and “reporting of bias” are not standard and not addressed in the different tools.

A closer look at the weighted scoring system implemented in the Trumble tool [30] and the Grid ULiège [28] reveals that criteria related to the evidence-based methodology are the most important, as they receive the highest weight factor. The Trumble tools gives equal weight to criteria related to usability and currency, while Grid ULiège gives lower weight to criteria related to usability. Banzi et al [3] based their tool on criteria from research on systematic review reporting methods and peer-reviewed medical journals’ policies. The tool was developed to check point-of-care information against their claims of being evidence-based [3,4], which clarifies focus on this topic. The eHealth Code of Ethics [18], the Banzi tool [3], and the 11-Point Quality Assessment Scale [26] focus on the evidence-based aspects but have little or no attention for the category “website design and usability,” which is related to the point-of-care aspect of information. Health information sources that are difficult to navigate will likely be used less since time constraints are an important barrier for health care professionals [2,37]. Therefore, the ideal tool should find the right balance between evidence-based methodology and usability-related criteria.

The Grid ULiège include detailed criteria, but the descriptions were sometimes unclear and seemed to contain overlapping items. Conversely, other tools [17-20,24,25,27,31] addressed multiple content aspects in only one criterion. Some tools were very concise in terms of the number of criteria [19,23] and seemed insufficient for a thorough evaluation of medical information, while others were too extensive and detailed and were therefore difficult to use [28,29]. These findings show that an adequate definition of criteria, together with a rational number of criteria, is indispensable for the usability of a tool.

For a few included tools [3,18,27,28,30,31], some criteria were excluded because they were considered inappropriate for the assessment of trustworthiness or not applicable in the current context (eg, criteria related to electronic commerce and marketing or drug-specific criteria) (see Multimedia Appendix 2). The criterion “breadth and volume” was excluded because it would disadvantage information sources that were designed for one pathology or treatment. Moreover, the specificity of an information source did not necessarily affect the quality, and small volume sources may contain useful information for practitioners.

### Practical Implications

As digitization is continuing in the health care sector and point-of-care information may play an increasingly important role in the daily practices of health care professionals, a valid evaluation tool for this kind of medical information is necessary. The usability of such a tool may depend on the user. Tools meant for health care professionals need to be short, whereas tools meant for external organizations that aim to validate information sources may be more comprehensive.

The current situation is problematic: There is no standard, valid tool available for health care professionals for a proper assessment of medical, point-of-care information. The use of the AGREE II instrument for the assessment of clinical guidelines was previously associated with enhanced guideline adoption: increased guideline endorsements, an increase in overall intentions to use guidelines, and an increase in overall quality of guidelines [8]. Therefore, the use of a tool for assessment of point-of-care information may improve the quality and use of this kind of information. Based on the results of this review, we suggest that such a tool should evaluate author-related information and evidence-based methodology. The items from the categories “website quality,” “website design and usability,” and “website interactivity” can be used to assess whether an information source is truly point-of-care information.

### Limitations

When we performed the literature search for this review, we noticed the absence of a common terminology for assessment of trustworthiness of point-of-care information. This is a limitation of this review that might have affected the output of the literature search. A broad search was needed to cover all tools used for different applications in medicine. Similarly, Kwag et al [4] noticed that point-of-care information summaries use different terms. We agree with their statement that a standard definition would be beneficial for the PubMed Mesh vocabulary.

A standard risk-of-bias assessment on each tool could not be performed, as no standard to assess this is currently available. Therefore, it was not possible to distinguish methodologically sound tools from those that are methodologically weak. However, each tool was checked for potential risk of bias in the developmental phase, such as lack of validity and reliability assessment.

### Conclusion

In conclusion, this systematic literature review identified 17 different tools for the assessment of the trustworthiness of point-of-care information. These tools encompass a variety of items, but to date, a standard, validated tool is nonexistent. The results of this review may contribute to the development of a standard tool, which may enhance the quality and trustworthiness of point-of-care information in the longer term.

## Appendix

Multimedia Appendix 1 Included tools and criteria.

Multimedia Appendix 2 Data summary.

## Abbreviations

AGREE II: Appraisal of Guidelines for Research and Evaluation
AMA: American Medical Association
CART: Completeness, Accuracy, Relevance, Timeliness
eHealth: electronic health
HON: health on the net
PRISMA: Preferred Reporting Items for Systematic Reviews and Meta-Analyses
QUEST: Quality Evaluation Scoring Tool
