# Supplementary material for: Tools to Assess the Trustworthiness of Evidence-Based Point-of-Care Information for Health Care Professionals: Systematic Review
Source: J Med Internet Res. 2020 Jan 17;22(1):e15415. doi: 10.2196/15415 (PMC6996752; doi:10.2196/15415)
Supplement: Multimedia Appendix 1 [file jmir_v22i1e15415_app1.docx]

# APPENDIX 1: Included Tools and Criteria

|  | A. Silberg criteria (4 items) |
| --- | --- |
| A1. | Authors and contributors, their affiliations, and relevant credentials are provided. |
| A2. | References and sources for all content should be listed clearly, with all relevant copyright information. |
| A3. | Website ownership should be fully disclosed, as should sponsorship, advertising, underwriting, commercial funding or potential conflicts of interests. |
| A4. | Dates that content was posted and updated should be indicated. |
|  | B. HONcode (8 items) |
| B1. | Any medical advice is only given by medically trained and qualified professionals (unless clearly stated otherwise). |
| B2. | Information is designed to support not replace the relationship between a patients and his/her physician. |
| B3. | The website respects confidentiality of data (incl. their identity) related to individual patients and visitors. |
| B4. | Information will be supported by clear references of source data. The date when a page was last modified is clearly displayed. |
| B5. | Any claims relating to benefits of a treatment, product or service will be supported by appropriate balanced evidence. |
| B6. | Information is provided in the clearest possible manner and provide contact addresses for those who seek further information or support. E-mailadres of webmaster is displayed on the website. |
| B7. | Support for the website is clearly identified, including commercial and non-commercial organizations that contributed funding, materials or services to the site. |
| B8. | If advertising is a source of funding, it will be clearly stated. Advertising is presented in a manner that facilitates differentiation between it and the original materials. |
|  | C. Kapoun criteria (5 items) |
| C1. | Page lists the author and institution that published the page and provides a way of contacting him/her. |
| C2. | Page lists author credentials and its domain is preferred (.edu; .gov; .org; .net). |
| C3. | Page provides accurate information with limited advertising and it is objective in presenting the information. |
| C4. | Page is current and updated regularly (as stated on page) and the links (if any) are also up to date. |
| C5. | It can view the information properly - not limited to fees, browser technology, or software requirement. |
|  | D. Sandvik scores (7 items) |
| D1. | Name and type of provider clearly stated. |
| D2. | Name and qualification of author clearly stated. |
| D3. | References given to scientific literature. |
| D4. | Date of publication or update clearly stated. |
| D5. | Clear invitation to comment or ask questions by email or link. |
| D6. | Information easily found by following links from home page. |
| D7. | Balanced information (not biased in favor of own products or services). |
|  | E. Gillois criteria (9 items) |
| E1. | Clear statement about objective. |
| E2. | Clear statement about context of development. |
| E3. | Clear statement on authorship. |
| E4. | Clear statement on financial relationships. |
| E5. | Information is based on some explicit evidence. |
| E6. | Evidence was easy to check (what's its level, is it applied according to the original study). |
| E7. | Ease of speed of access to the site. |
| E8. | Speed and conviviality of interface. |
| E9. | Interactivity: possibility to communicate with the authors to get explanations or any other information. |
|  | F. Joubert criteria (8 items) |
| F1. | Authority of source (database provider, website provider, organization). |
| F2. | Coverage of source (overview and possibility of multiple sources and interrelationships). |
| F3. | Accessibility of source (restrictions to access, different languages, speed of information accessibility). |
| F4. | Coverage of content (description, type, time coverage, context, intended audience). |
| F5. | Currency of content (frequency and type of updating). |
| F6. | Ease of exploration (information retrieval, navigation). |
| F7. | Clear display (adaptive ranking, significant number of record, customizable period of research …). |
| F8. | Availability of help (support for training, online help, introduction to system's behavior). |
|  | G. AMA principles (13 items*) |
| G1. | Website ownership should be clearly indicated. |
| G2. | Site should provide information about the platform and browser that permit optimal viewing. |
| G3. | Information about restrictions on access to content, registration and password protection should be provided and easy to find. |
| G4. | Funding or other sponsorship for any specific content should be clearly indicated (and comply with principles for advertising). |
| G5. | Content should be reviewed for quality (including originality, accuracy and reliability) before posting. |
| G6. | The dates that content is posted or revised and updated should be clearly indicated. |
| G7. | Source for specific content should be clearly identified (i.e. author byline or names of individual, organization …). Affiliations and relevant financial disclosures for authors and content producers should be clearly indicated. |
| G8. | Intrasite content links should be reviewed before posting and maintained and monitored. |
| G9. | Sites should not prevent viewers from returning to a previous site. |
| G10. | If content can be downloaded, instructions should be available regarding procedure and software. |
| G11. | Features that facilitate use of the site should be provided and easy to find (help function, FAQ, customer service ...). |
| G12. | Principles for advertising and sponsorship. |
| G13. | Principles for privacy and confidentiality. |
|  | H. e-Health Code of Ethics (17 items*) |
| H1. | Who owns the site (candor). |
| H2. | Purpose of the site (candor). |
| H3. | Conflict of interest (financial, professional, personal or other) (candor). |
| H4. | Forthright in all content used to promote the sale of health product or services (honesty). |
| H5. | Forthright in any claims about the efficacy, performance, or benefits of products or services (honesty). |
| H6. | Evaluate information rigorously and fairly, including information used to describe products or services (quality, accuracy). |
| H7. | Provide information that is consistent with the best available evidence (quality, based on evidence). |
| H8. | Assure that when personalized medical care or advice is provided that care or advice is given by a qualified practitioner (quality, expertise). |
| H9. | Indicate clearly whether information is based on scientific studies expert consensus, or professional or personal experience or opinion (quality). |
| H10. | Acknowledge that some issues are controversial and when that is the case make good faith efforts to present all reasonable sides in a fair and balanced way (quality). |
| H11. | Information and services must be easy for use for consumers to understand and use. Sites should present information and describe products or services (quality). |
| H12. | Sites that provide information primarily for educational or scientific purpose should guarantee the **independence of their editorial policy** and practices by assuring that only the site's content editors determine editorial content and have the authority to reject advertising that they believe is inappropriate (quality). |
| H13. | Consumers have a right to expect that the information they receive is up to date (date of publication, review, updates should be mentioned) (quality). |
| H14. | Describe what sources the site or content provider has used, with reference or links to those sources (quality). |
| H15. | How the site evaluates content and what criteria are used to evaluate content, including on what basis the site decides to provide specific links to other sites or services. |
| H16. | Insist that current or prospective sponsors not influence the way search results are displayed for specific information on key words or topics. |
| H17. | Provide meaningful opportunity for users to give feedback to the site (accountability). |
|  | I. Jiang criteria (7 items) |
| I1. | Source of site: site published by professionals (dentists, orthodontists etc.) |
| I2. | Information is intended for patient education or for academic use for professionals. |
| I3. | Authors or contributors had relevant credentials. |
| I4. | References related to the content were listed. |
| I5. | Purpose of site was clearly stated, ownership with any sponsorship, advertising, potential conflict of interest or commercial funding were fully disclosed. |
| I6. | Accuracy of information "the judgment as to whether the site information was correct, questionable or incorrect was determined". |
| I7. | The date that the content of the website was posted or updated was given. |
|  | J. Grid ULiège (24 items*) |
| J1. | Detailed information / size of the site (content). |
| J2. | Online access to full texts of documents (content). |
| J3. | Relevance of links to other sites / links still active? (Content). |
| J4. | Short description of related/linked sites (content). |
| J5. | Useful presentation and relevance of illustrations and animations (content). |
| J6. | Presentation of the inclusion and exclusion criteria of data (content). |
| J7. | Level of evidence (respect of principles of EBM) (content). |
| *J8.* | *Logical organization of information (content).* |
| *J9.* | *Disclaimer: Provided description of the purpose of the site, its scope, authority and timeliness of the information? Mention of sources of information (to ensure accuracy and absence of plagiarism)? Presence of clarification that this is a general health information and not a medical consultation? Is there clarity about the fact that only the doctor and other professionals in the health care sector can advise patients? (content).* |
| J10. | Does website describe the aim of the information (not personalized medical advice)? |
| J11. | Name and logo of institute should be presented (ownership) (trustworthiness). |
| *J12.* | Name and credentials of authors should be reported (trustworthiness). |
| *J13.* | Source of information is clear (trustworthiness). |
| *J14.* | Date of creation or last update is reported (trustworthiness). |
| *J15.* | Is there a editorial team to review content + list names (trustworthiness). |
| *J16.* | Is there a webmaster? (Trustworthiness). |
| *J17.* | Who finances the site / COI author (trustworthiness). |
| *J18.* | Absence of conflicts of interest (trustworthiness). |
| *J19.* | Target audience is clear (+ type of access: free or paying) (trustworthiness). |
| *J20.* | Readability of information (trustworthiness). |
| *J21.* | Clear navigation (navigation). |
| *J22.* | Easiness of search (navigation). |
| *J23.* | Website loads quickly (navigation). |
| *J24.* | Hyperlinks are checked (navigation). |
| *J25.* | Possibility to contact webmaster/authors (navigation). |
| *J26.* | Presence of a help page? |
| *J27.* | Presence of a discussion forum? |
|  | K. CART (4 items) |
| K1. | Completeness |
| K2. | Accuracy |
| K3. | Relevance |
| K4. | Timeliness |
|  | L. Trumble Tool (6 items*) |
| L1. | Authorship: transparency, credentials listed, are entries peer reviewed |
| L2. | Updating: how often new topics added, how often records updated/revised? |
| L3. | Bias: did you see bias, please describe these |
| L4. | usability: easy of navigation, printing, other output - downloading, description, help screens |
| L5. | Does it grade the evidence? |
| L6. | Is content referenced? |
|  | M. Banzi tool (10 items*) |
| M1. | Clear indication of the author(s) of a specific content. |
| M2. | Detailed description of the procedures aimed at assessing and ensuring the scientific quality of output (review process by external peer reviewers and/or editors). |
| M3. | Content updating within two years. |
| M4. | A formal policy on authors' commercial conflict of interests is implemented and this information is reported. |
| M5. | To what extend commercial support and advertising are accepted in the content development policy. |
| M6. | Contents are written on basis of specific systemic literature search based on explicit search strategies aimed at identifying relevant and valid articles, OR if systematic tracking of the relevant and valid articles is utilized. |
| M7. | Content is preferably written on the basis of systematic reviews, rather than other publications. |
| M8. | Use of standard and transparent methods to assess articles' validity. |
| M9. | A formal system is implemented to grade the level of evidence. |
| M10. | Statements based on experts' opinions are easily recognizable compared to study data and results. |
|  | N. OncoRx-IQ (11 items*) |
| N1. | Does the database contain a help feature? |
| N2. | Does the database contain a site map, index or menu system that facilities navigation? |
| N3. | Does the database state its source(s) of funding? |
| N4. | Does the database contain any evidence of bias (e.g. advertisements)? |
| N5. | Does the database state the name(s) and credential(s) of the authors(s)? |
| N6. | Does the database state how it is reviewed for information accuracy? |
| N7. | How does the database cite the source(s) used to compile the drug interaction information? |
| N8. | Are there links to other sources (e.g. PubMed abstracts and/or original articles, online drug databases) to verify the drug information in the database. |
| N9. | The drug database clearly states the types of personal information it collects and how the information is used. |
| N10. | The drug database states clearly who the target audience are. |
| N11. | The drug database specifies that the information is NOT meant to replace the advice of a healthcare professional. |
|  | O. 11 Point Quality Assessment Scale (11 items) |
| O1. | In-line references for treatment recommendations. |
| O2. | In-line references for diagnostic recommendations. |
| O3. | Policy indicating steps to find new evidence. |
| O4. | Policy indicating the rating of research evidence. |
| O5. | Policy indicating the grading of evidence. |
| O6. | Date stamping of individual chapters. |
| O7. | Indication of a schedule for updating chapters. |
| O8. | "New evidence" tabs for individual chapters / topics. |
| O9. | User alerts for new evidence according to user discipline. |
| O10. | User alerts for nex evidence according to individual topic. |
| O11. | Federated search of content and external evidence source. |
|  | P. Aslani criteria (10 items) |
| P1. | Identify who wrote the material, who are responsible for the professional and scientific edition and review of the website materials. |
| P2. | Clarify who is responsible for the site and its information. |
| P3. | What is the funding source of the website and advertising policy. |
| P4. | Present a clear statement of the purpose. |
| P5. | Clearly state the original source of information if it is collected from other websites or sources. |
| P6. | Readability of test, use of images, graphs, video and so on. |
| P7. | Easily finding the information on the site or other sites by search engine or links. |
| P8. | Describing web site liability and privacy statement. |
| P9. | Ways to contact the site owner (email, chat). |
| P10. | Dates of update or review. |
|  | Q. QUEST (6 items) |
| Q1. | Authorship (name and qualification). |
| Q2. | Attribution (references to sources / scientific studies). |
| Q3. | Conflict of interest. |
| Q4. | Currency. |
| Q5. | Complementarity (support of the patient-physician relationship. |
| Q6. | Tone (includes title) (balanced/cautious support of claims, includes statements of limitations and/or contrasting findings. |
|  |  |
|  | (*) only items relevant for the assessment of trustworthiness of point-of-care resources are considered in this table. The tool contained more items in its original form. |
|  | **AMA**: principles for e-commerce were not included in this table. |
|  | **e-Health Code of Ethic**s: Items related to Informed Consent, Privacy, Professionalism in Online Healthcare and Responsible Partnering were not included in this table |
|  | **Grid developed by University of Liege:** Other items, not included in this table: items of **'content'** (coverage of source, online access to full texts, relevance of external links, brief description of links, presence of useful illustrations, clear description of included and excluded information, are principles of EBM respected (levels of evidence), how is information organized. |
|  | Items of **'trustworthiness'**: Metadata (description of the information provided in the web document in the 'source code' of the page): Completeness and quality of the information introduced at the metadata level? Reference to authoritative classifications (Medical Subject Headings or MeSH, Library of Congress)? Participation in institutional projects like the Dublin Core (DC metadata)? |
|  | Items of **'navigation'**: Easy understanding of orientation buttons: Meaningful icons? General index, site map and / or help page: Presence of a general index of all the HTML documents present on the site? Presence of the site plan (graphic or textual)? Presence of a specific glossary for acronyms or special characters used? "What's new" section: Presence of a "what's new" section to quickly see what's new on the site? In case of modification of the structure of the site, links between the old HTML documents and the new ones: Link between the old and the new URL of a document that would have been moved within the site to avoid broken links? Forums, "Ask an expert": Possibility to converse with a specialist of the specialty? |
|  | Items of **'visual presentation**': Site design and colors used: Sobriety of colors? Airy presentation? Readability of the text: Ease of reading on the screen? Choice of characters? Harmony between the background color and the characters? Presentation of information adapted to the target audience? Quality and speed of illustrations loading: Speed of loading images? Print quality: Text format? Readability of the prints especially according to the colors? Complete match between what is read on screen and what is printed? |
|  | Items of '**accessibility**': Presence in the main directories and search tools: Site identifiable by the main directories and search engines (French, European and global search engines)? Intuitive address: Significant identification of the address of the site? Easy tracking? |
|  | **Trumble Tool:** Items not included in this table: 'Inclusion': is this point-of-care info? 'General Information': Subscription (free, individual, institutional), cost, Simultaneous users, Target audience of marketing, Marketing (claims, does product match the claims). 'Content': Scope (Volume, breadth, drug information, future expansion). Patient Handouts, CE credits, Practice guidelines. 'Searching': types of searches available. 'Results': Type of questions answered, presentation of results, summary of evidence. Other features: customization, integration of technologies, unique or useful features, coming features.  **Banzi tool:** Volume (Breadth of diseases covered) was not included in the table. |
|  | **Onco-Rx:** drug specific criteria were not included in the table: 1.Is/are the correct interaction effects provided for the drug pair? 2. is/are the correct mechanism(s) of interaction provided for the above drug pair? 3. Is/are the correct evidence(s) (e.g.: care reports, animal, human or in vitro studies- provided for the interaction concerning the drug pair? Is /are the appropriate recommendation(s) provided to manage the interaction for the drug pair? Can the database search for drug interaction using chemotherapy regimen acronyms (e.g. BEACOPP, ESHAP, R-CHOP)? Can the database search for more than one drug interaction simultaneously? Is the probability AND the severity of the drug interactions stated? The drug database clearly states the types of drug-related information it provides? |
