# Supplementary material for: Tools to Assess the Trustworthiness of Evidence-Based Point-of-Care Information for Health Care Professionals: Systematic Review
Source: J Med Internet Res. 2020 Jan 17;22(1):e15415. doi: 10.2196/15415 (PMC6996752; doi:10.2196/15415)
Supplement: Multimedia Appendix 2 [file jmir_v22i1e15415_app2.docx]

Table 2. Data summary.

| **General criteria** | **linked criteria (see Appendix 1 for full description)** | **A. Silberg scores** | **B. HONcode** | **C. Kapoun criteria** | **D. Sandvik scores** | **E. Gillois criteria** | **F. Joubert criteria** | **G. AMA principles** | **H. e-Health Code of Ethics** | **I. Jiang criteria** | **J. Grid Uliège** | **K. CART** | **L. Trumble Tool** | **M. Banzi Tool** | **N. OncoRx-IQ** | **O. 11 Point Quality Assessment scale** | **P. Aslani criteria** | **Q. Quest** |
| --- | --- | --- | --- | --- | --- | --- | --- | --- | --- | --- | --- | --- | --- | --- | --- | --- | --- | --- |
| **1.** **AUTHOR RELATED INFORMATION** |  |  |  |  |  |  |  |  |  |  |  |  |  |  |  |  |  |  |
| Author's name and affiliation are reported | A1, C1, D2, E3, G7, J12, L1, M1, N5, P1, Q1 | x |  | x | x | x |  | x |  |  | x |  | x | x | x |  | x | x |
| Authors are qualified or authorative on the topic; Authors credentials are reported | A1, B1, C2, D2, H8, I1, I3, J9, J12, L1, N5, Q1 | v | x | x | v |  |  |  | x | xx | xx |  | x |  | v |  |  | v |
| Author's COI is reported | A3, E4, G7, H3, I5, J9, J17, J18, M4, Q3 | v |  |  |  | x |  | v | x | v | xxx |  |  | x |  |  |  | x |
| Independence of editors is guaranteed | H12 |  |  |  |  |  |  |  | x |  |  |  |  |  |  |  |  |  |
| **2. EVIDENCE BASED METHODOLOGY** |  |  |  |  |  |  |  |  |  |  |  |  |  |  |  |  |  |  |
| Inclusion and exclusion criteria for studies /data are reported | J6 |  |  |  |  |  |  |  |  |  | x |  |  |  |  |  |  |  |
| Information is designed to support, not replace, the relationship between a patients and his/her physician | B2, J9, N11, Q5 |  | x |  |  |  |  |  |  |  | x |  |  |  | x |  |  | x |
| References to source data or info | A2, B4, E6, H9, H14, H15, I4, J9, J13, L6, N7, N8, O1, O2 P5, Q2 | x | x |  |  | x |  |  | xxx | x | xx |  | x |  | xx | xx | x | x |
| Cite expert opinions | H9, M10 |  |  |  |  |  |  |  | v |  |  |  |  | x |  |  |  |  |
| Content is based on evidence | B5, D3, E5, H7 |  | x |  | x | x |  |  | x |  |  |  |  |  |  |  |  |  |
| Content is current and actual (publication data/ updates are reported) | A4, B4, C4, D4, F5, G6, H13, I7, J9, J14, K4, L2, M3, O6, O8, O7, P10, Q4 | x | v | x | x |  | x | x | x | x | xx | x | x | x |  | xxx | x | x |
| Content is accurate or objective or transparant | C3, D7, H4, H5, H10, I6, K2, N4, Q6 |  |  | x | x |  |  |  | xxx | x |  | x |  |  | x |  |  | x |
| Content is complete (detailed info, coverage of the source, size of the site) | F2, F4, J1, K1, |  |  |  |  |  | xx |  |  |  | x | x |  |  |  |  |  |  |
| Content is relevant | K3 |  |  |  |  |  |  |  |  |  |  | x |  |  |  |  |  |  |
| Literature search and surveillance | M6, O3 |  |  |  |  |  |  |  |  |  |  |  |  | x |  | x |  |  |
| Systematic reviews are preferred above primary studies | M7 |  |  |  |  |  |  |  |  |  |  |  |  | x |  |  |  |  |
| Critical appraisal of evidence / transparant quality assessments | H6, M8, O4 |  |  |  |  |  |  |  | x |  |  |  |  | x |  | x |  |  |
| Formal grading of evidence | J7, L5, M9, O5 |  |  |  |  |  |  |  |  |  | x |  | x | x |  | x |  |  |
| Possible bias is reported | L3 |  |  |  |  |  |  |  |  |  |  |  | x |  |  |  |  |  |
| Content is (externally) reviewed or peer reviewed | G5, J15, L1, M2, N6 |  |  |  |  |  |  | x |  |  | x |  | x | x | x |  |  |  |
| **3. WEBSITE QUALITY** |  |  |  |  |  |  |  |  |  |  |  |  |  |  |  |  |  |  |
| Clear purpose | E1, H2, I2, I5, J9, J10, P4 |  |  |  |  | x |  |  | x | xx | xx |  |  |  |  |  | x |  |
| Clear statement about the context of development | E2 |  |  |  |  | x |  |  |  |  |  |  |  |  |  |  |  |  |
| Target audience described | F4, J19, N10 |  |  |  |  |  | x |  |  |  | x |  |  |  | x |  |  |  |
| Transparant ownership | A3, B1, D1, F1, G1, H1, I5, J10, P2 | x | x |  | x |  | x | x | x | v | x |  |  |  |  |  | x |  |
| Short description of related / linked sites | J4 |  |  |  |  |  |  |  |  |  | x |  |  |  |  |  |  |  |
| Financial information (financial disclosures, advertising & sponsorship policy,… reported) | A3, B7, B8, G4, G12, H16, I5, J17, J18, M5, N3, P3 | v | xx |  |  |  |  | xx | x | v | xx |  |  | x | x |  | x |  |
| Liability and privacy statement, respect of confidentiality of data | B3, G13, N9, P8 |  | x |  |  |  |  | x |  |  |  |  |  |  | x |  | x |  |
| Webmaster present | J16 |  |  |  |  |  |  |  |  |  | x |  |  |  |  |  |  |  |
| **4. WEBSITE DESIGN AND USABILITY** |  |  |  |  |  |  |  |  |  |  |  |  |  |  |  |  |  |  |
| Readability / (info on) optimal viewing and logical organisation of content | B6, E8, G2, J8, J20, P6, |  | v |  |  | x |  | x |  |  | xx |  |  |  |  |  | x |  |
| Useful presentation and relevance of illustrations and animations | J5 |  |  |  |  |  |  |  |  |  | x |  |  |  |  |  |  |  |
| Ease of use, search and navigation | C5, D6, E7, F6, F7, G8, H11, J2, J21, J22, J23, J24, L4, N2, O11, P7 |  |  | x | x | x | xx | x | x |  | xxxxx |  | x |  | x | x | x |  |
| Information on access (restrictions, payable, information on how to login…) | F3, G3, J19 |  |  |  |  |  | x | x |  |  | x |  |  |  |  |  |  |  |
| Intersite navigation | G9, J3 |  |  |  |  |  |  | x |  |  | x |  |  |  |  |  |  |  |
| Downloading content (possibility, instructions, speed) | G10, L4 |  |  |  |  |  |  | x |  |  |  |  | x |  |  |  |  |  |
| Help and support function | F8, G11, J26, L4, N1 |  |  |  |  |  | x | x |  |  | x |  | x |  | x |  |  |  |
| **5. WEBSITE INTERACTIVITY** |  |  |  |  |  |  |  |  |  |  |  |  |  |  |  |  |  |  |
| Possibility to contact site owner or authors, to ask questions or give feedback | B6, C1, D5, E9, H17, J25, J27, P9 |  | x | v | x | x |  |  | x |  | xx |  |  |  |  |  | x |  |
| User alerts for new evidence/ next evidence according to user discipline/individual topic | O9, O10 |  |  |  |  |  |  |  |  |  |  |  |  |  |  | xx |  |  |

x: item mentioned in the tool; v: item covered by the tool, but not separately defined as an item.

*Note: The 17 included tools cover 156 criteria in total. To synthesize these, they were reformulated and linked to 36 general criteria (column 1), divided into five categories. The codes in column 2 refer to the original wording of the criterion in Appendix 1.*
